# Prediagnostic markers of insulin resistance and prostate cancer risk and death: A pooled study

**DOI:** 10.1002/cam4.6004

**Published:** 2023-04-27

**Authors:** Sylvia H. J. Jochems, Josef Fritz, Christel Häggström, Pär Stattin, Tanja Stocks

**Affiliations:** ^1^ Department of Clinical Sciences Lund Lund University Lund Sweden; ^2^ Department of Surgical Sciences Uppsala University Uppsala Sweden; ^3^ Department of Translational Medicine Lund University Malmö Sweden; ^4^ Institute of Medical Statistics and Informatics, Medical University of Innsbruck Innsbruck Austria; ^5^ Northern Registry Centre, Department of Public Health and Clinical Medicine Umeå University Umeå Sweden

**Keywords:** insulin resistance, prospective studies, prostatic neoplasms

## Abstract

**Background:**

Insulin resistance has been shown to be related to a higher risk of several cancers, but the association with prostate cancer (PCa) has been inconsistent.

**Methods:**

We investigated prediagnostic markers of insulin resistance in men in four cohorts in Sweden, in relation to PCa risk (total, non‐aggressive and aggressive) and PCa death using multivariable‐adjusted Cox regression. The number of men, PCa cases and PCa deaths was up to 66,668, 3940 and 473 for plasma glucose and the triglyceride‐glucose (TyG) index, and up to 3898, 586 and 102 for plasma insulin, glycated haemoglobin (HbA1c) and leptin.

**Results:**

Higher HbA1c was related to a lower risk of non‐aggressive PCa but no significant associations were found for insulin resistance markers with the risk of aggressive or total PCa. In PCa cases, higher glucose and TyG index were related to a higher risk of PCa death (hazard ratio [HR] per higher standard deviation, 1.22, 95% CI 1.00–1.49 and 1.24, 95% CI 1.00–1.55), which further increased when restricting the analyses to glucose and TyG index measures taken <10 years before the PCa diagnosis (HR, 1.70, 95% CI 1.09–2.70 and 1.66, 95% CI 1.12–2.51). No associations were observed for other markers in relation to PCa death.

**Conclusions:**

The results of this study showed no associations of insulin resistance markers with the risk of clinically relevant PCa, but higher glucose and TyG index were associated with poorer survival from PCa. The lack of association for other insulin resistance markers may be due to their smaller sample size.

## 1 INTRODUCTION

Insulin resistance is related to a higher risk of several forms of cancer^1^ but in relation to prostate cancer (PCa), the commonest cancer in men in high‐income countries, evidence remains unclear. Some markers of insulin resistance, including elevated plasma glucose and glycated haemoglobin (HbA1c), a marker of long‐term blood glucose level, have shown lower risks in relation to PCa,^2^, ^3^, ^4^, ^5^ especially localised PCa. However, these findings could be due to delayed detection of PCa in men with obesity and type 2 diabetes mellitus (T2DM), both common conditions in men with insulin resistance and also related to lower levels of prostate‐specific antigen (PSA) and potentially less PCa screening.^6^, ^7^, ^8^ Markers of insulin resistance have been related to a higher risk of aggressive PCa in some studies whereas other studies found no association.^9^, ^10^, ^11^

Insulin resistance markers may be involved in the progression of PCa. This is supported by a few studies that have shown a higher risk of metastases or PCa death among men with elevated glucose, insulin and HbA1c levels.^12^, ^13^, ^14^, ^15^, ^16^, ^17^, ^18^ Moreover, men diagnosed with PCa who have T2DM have a higher risk of all‐cause mortality and PCa‐specific mortality compared to patients without T2DM.^19^ Chronic inflammation and higher levels of insulin and insulin‐like growth factor‐1 (IGF‐1) are some of the proposed mechanisms behind these associations.^12^, ^13^

In this study, we investigated prediagnostic markers of insulin resistance, including glucose, insulin, HbA1c, leptin and the triglyceride‐glucose (TyG) index—an indicator of insulin resistance,^20^ in relation to the risk of PCa and PCa death, in total and for non‐aggressive and aggressive PCa separately.

## 2 METHODS

### 2.1 Study participants

We included information from health examinations of men conducted in 1974–2016 in four Swedish cohorts. These were the Västerbotten Intervention Programme (VIP),^21^, ^22^ the Northern Sweden Monica study,^23^, ^24^ the Malmö Diet and Cancer Study (MDCS)^25^, ^26^ and the Malmö Preventive Project (MPP).^27^, ^28^ At the health examination, participants provided a blood sample from which levels of one or several markers of insulin resistance (plasma glucose, insulin, HbA1c and leptin) were measured. In this study, we only included men who had fasted at least 8 h before the blood draw. In the VIP, HbA1c and leptin were measured from frozen–thawed samples in a nested case–control study.^29^ Insulin from the MPP and all markers except HbA1c in the MDCS were measured in a random sample of the original cohort. Plasma triglyceride levels were measured in all cohorts and were used to calculate the TyG index according to the formula: ln [triglycerides (mg/dL) × plasma glucose (mg/dL)/2].^20^ Data of measured body mass index (BMI, kg/m^2^), and questionnaire information on smoking and history of diabetes were also collected from the baseline examination in the cohorts. Detailed descriptions of the protocols for measuring the markers of insulin resistance in each cohort of our pooling have been previously published.^24^, ^30^, ^31^, ^32^, ^33^

### 2.2 Follow‐up of participants

By use of the unique personal identification number assigned to all Swedish residents, we followed up study participants in Swedish nation‐wide registers until 31 December 2016. The Swedish Cancer Register^34^ was used to identify PCa diagnoses (ICD‐7177) and other cancers, and the Swedish Cause of Death Register,^35^ which has an 86% concordance with medical records for deaths due to PCa, was used to determine the cause and date of death.^36^ Participants were also linked to the Total Population Register to obtain information on emigration, the Longitudinal Integration Database for Health Insurance and Labour Market Studies for information on birth country and socioeconomic factors, and the Patient Register which provided data on in‐patient care that we used to calculate the Charlson comorbidity index.^37^ Information from the National Prostate Cancer Register of Sweden^38^ was obtained for PCa characteristics at the time of diagnosis and for primary treatment. PCas were classified as aggressive or non‐aggressive. As suggested by Hurwitz et al.^39^ PCas with any one of T4, N1, M1 or Gleason score ≥8 were categorised as aggressive, and in addition, we also included cases with a diagnostic PSA level of 50 ng/mL or higher in the aggressive category. Hurwitz et al. did not include diagnostic PSA level as risk categorisation criteria due to incomplete data.^39^ PCas without any of these characteristics were classified as non‐aggressive.

### 2.3 Statistical analysis

All markers were standardised (z‐transformed) separately by cohort to account for different measurement methods. The marker's interrelationships and correlations with BMI were calculated by Spearman's partial rank correlation adjusted for age. We applied Cox regression with age as time scale to calculate hazard ratios (HRs) with 95% confidence intervals (CIs) for PCa risk and PCa death across tertiles and per standard deviation (SD) higher level of each insulin resistance marker. Cohort‐specific tertile cut‐points are shown in Table S1. We adjusted the Cox model for age at study entry (continuous) and stratified on cohort and year of birth (<1935, 1935 to 1939, 1940 to 1944, 1945 to 1949, ≥1950). In a fully adjusted analysis, we additionally adjusted for history of diabetes (yes, no, missing), birth country (Sweden‐born and both parents Sweden‐born, Sweden‐born and one parent Sweden‐born, Sweden‐born and both parents born abroad, born abroad, missing), education (seven categories or missing), baseline BMI (<25, 25–29.9, ≥30 kg/m^2^) and baseline smoking status (never, former, or current smoker, or missing). Because HbA1c and leptin levels in the VIP originated from a case–control study (nested within a cohort), the association of these markers with PCa incidence and death was calculated expressed as odds ratios (ORs) computed by logistic regression adjusted for baseline age, cohort, year of birth, country of birth, history of diabetes, education, body mass index and smoking status. Besides the VIP, the MDCS also contributed data to the analysis of HbA1c and leptin. In this cohort, HRs and ORs were very similar for HbA1c and leptin levels (Table S2), which together with the investigation of a rare outcome in this study, supported the use of logistic regression in the pooling.

In addition to full‐cohort analyses from baseline to death, we conducted case‐only survival analyses to investigate insulin resistance markers in relation to PCa death. These analyses were performed in PCa cases with clinical characteristics information available from the National Prostate Cancer Register. We applied Cox regression with time since diagnosis as time scale and adjusted for the same variables and stratified the Cox model similarly to the full‐cohort analysis. Exceptions were done for age at baseline, which was not adjusted for, and education level, which instead of the baseline level regarded the time closest to the PCa diagnosis. Additionally, case‐only analyses were adjusted for age at diagnosis (continuous), time since baseline (continuous), Charlson comorbidity index (none, mild, severe), primary treatment (conservative, curative, non‐curative, missing) and PCa category (aggressive/non‐aggressive). Sensitivity analyses were performed for glucose and TyG index levels whereby smokers, obese men, diabetic men, or men with severe or any comorbidities were excluded.

Approximately 40% of men had repeated measurements for glucose and triglycerides. Because long‐term variation and random measurement error of an exposure dilute its association with disease, we corrected the HRs of glucose and the TyG index for the regression dilution ratio (RDR) using the equation HR_corrected_ = exp(log[HR_original_]/RDR).^40^ The RDR was 0.40 for glucose and 0.50 for the TyG index in the full cohort, and 0.48 for glucose and 0.53 for the TyG index in cases only.

We tested the proportional hazards assumption with Schoenfeld residual statistics and by visual inspection of the hazard curves, which indicated no violation of the assumption. Statistical tests were two‐sided and were performed in STATA 15.1 (StataCorp LLC). We set the significance level to *p* < 0.05.

## 3 RESULTS

Table 1 shows the baseline characteristics of the 68,147 men included in the study. On average, participants were 47.3 (SD 10.3) years old at baseline. The number of men with information available for the insulin resistance markers varied and was the largest for glucose (*n* = 66,668) and TyG index (*n* = 56,897) and ranged between 2207 and 3898 for the other insulin resistance markers. Correlation coefficients between the markers, and between the markers and BMI, are shown in Table S3. All correlations were positive and ranged between 0.25 and 0.48.

**TABLE 1:** Baseline characteristics of the 68,147 men in the study according to cohort.

Characteristic	VIP^a^ (*N* = 48,371)	MONICA (*N* = 2642)	MDCS (*N* = 2229)	MPP (*N* = 14,905)
Year, range (median)	1986–2016 (1999)	1986–2014 (1994)	1991–1995 (1993)	1974–2006 (1980)
Age, years, mean (SD)	46.6 (8.9)	48.6 (13.9)	57.6 (6.0)	47.7 (12.9)
Year of birth, *n* (%)				
<1940	5549 (11)	821 (31)	1615 (72)	9447 (63)
1940–1949	11,354 (23)	572 (22)	614 (28)	5458 (37)
1950–1959	12,453 (27)	513 (19)	—	—
≥1960	19,015 (39)	736 (28)	—	—
Markers of insulin resistance^b^				
Glucose, mmol/L, mean (SD)	5.5 (0.8)	5.3 (0.7)	5.2 (0.7)	5.0 (0.6)
Triglycerides, mmol/L, mean (SD)	1.5 (0.7)	1.5 (0.8)	1.5 (0.7)	1.4 (0.7)
TyG index, mean (SD)^c^	8.6 (0.5)	8.6 (0.5)	8.5 (0.5)	8.5 (0.5)
Insulin, mLU/L, mean (SD)	—	—	7.5 (3.7)	8.0 (4.7)
HbA1c, %, mean (SD)	4.4 (0.5)	—	4.8 (0.5)	—
Leptin, ng/mL, mean (SD)	4.0 (1.7)	—	2.5 (0.8)	—
Diabetes, *n* (%)^d^				
No	47,229 (98)	2567 (97)	1801 (81)	14,147 (95)
Yes	877 (2)	51 (2)	76 (3)	758 (5)
Missing	265 (<1)	24 (1)	352 (16)	—
Body mass index, kg/m^2^, mean (SD)	26.5 (3.8)	26.6 (3.8)	26.2 (3.5)	25.1 (3.5)
Body mass index, kg/m^2^, *n* (%)				
<25	18,385 (38)	988 (38)	882 (40)	8087 (54)
25–29.9	22,664 (47)	1221 (46)	1075 (48)	5626 (38)
≥30	7322 (15)	433 (16)	272 (12)	1192 (8)
Smoking status, *n* (%)^d^				
Never smoker	30,134 (62)	1403 (53)	669 (30)	5268 (35)
Former smoker	10,080 (21)	806 (31)	919 (41)	3107 (21)
Current smoker	7310 (15)	425 (16)	575 (26)	6484 (44)
Missing	847 (2)	8 (<1)	66 (3)	—
Education, *n* (%)^e^				
Pre‐upper secondary school <9 y	5448 (11)	523 (20)	667 (30)	4384 (29)
Pre‐upper secondary school 9 y	8327 (17)	437 (17)	114 (5)	971 (7)
Max. 2 y upper secondary school	18,595 (39)	854 (32)	576 (26)	3571 (24)
3 y upper secondary school	5932 (12)	354 (13)	429 (19)	2550 (17)
Post‐upper secondary school <3 y	5331 (11)	258 (10)	217 (10)	1181 (8)
Post‐upper secondary school ≥3 y	4680 (10)	204 (8)	223 (10)	1493 (10)
Missing	58 (<1)	12 (<1)	3 (<1)	755 (5)
Country of birth, *n* (%)^f^				
Born in Sweden with both parents born in Sweden	43,687 (90)	2314 (88)	1899 (85)	12,306 (83)
Other	4684 (10)	328 (12)	330 (15)	2599 (17)

Abbreviations: MDCS, Malmö Diet and Cancer Study; MONICA, Northern Sweden Monica Study; MPP, Malmö Preventive Project; SD, standard deviation; VIP, Västerbotten Intervention Programme.

^a^ In the VIP, HbA1c and leptin information originated from a nested case–control study^29^.

^b^ The 76,510 men in the study had information on at least one insulin resistance marker measured in a fasting state. The number of men with complete information was for glucose 66,668; triglycerides 58,102; TyG index 56,897; insulin 3898; HbA1c 2881; and leptin 2364.

^c^ TyG index was calculated as ln[triglycerides (mg/dL) × plasma glucose (mg/dL)/2].

^d^ Determined from questionnaires.

^e^ Determined from the Swedish longitudinal integration database for health insurance and labour market studies.

^f^ Determined from the Swedish Multi‐generation Register, which has virtually complete coverage of first‐degree biological family for individuals born in or after 1932, registered in Sweden in 1961 or later.

Altogether, 4016 men received a PCa diagnosis during follow‐up of which 3761 had clinical characteristics information available from the National Prostate Cancer Register, which was used for categorisation of PCas as non‐aggressive (*n* = 2728) or aggressive (*n* = 1033) (Table 2). In fully adjusted models, HbA1c was negatively associated with non‐aggressive PCa (OR per higher SD, 0.86, 95% CI 0.75–0.99), and such negative association was near significant also for TyG index (RDR corrected HR per higher SD, 0.88, 95% CI 0.81–1.00). No other insulin resistance marker reached a significant association with PCa incidence or death in the full cohort followed from baseline (Table 3).

**TABLE 2:** Clinical characteristics of the 3761 prostate cancer cases identified in the National Prostate Cancer Register of Sweden.

Characteristic	Non‐aggressive prostate cancer^a^ (*n* = 2728)	Aggressive prostate cancer^a^ (*n* = 1033)
Year of diagnosis, range (median)	1998–2016 (2009)	1998–2016 (2007)
Age at diagnosis, years, mean (SD)	67.2 (6.7)	70.9 (7.3)
Charlson comorbidity index, *n* (%)		
0 (no comorbidity)	2346 (86)	868 (84)
1 (mild comorbidity)	218 (8)	93 (9)
≥2 (severe comorbidity)	164 (6)	72 (7)
Local clinical tumour stage, *n* (%)		
T1	1719 (63)	—
T2	845 (31)	527 (51)
T3	136 (6)	413 (40)
T4	—	62 (6)
Missing	28 (1)	31 (3)
Lymph node metastasis, *n* (%)		
N0	519 (19)	182 (18)
N1	—	129 (12)
Nx	2209 (81)	722 (70)
Bone metastasis, *n* (%)		
M0	1695 (62)	548 (53)
M1	—	319 (31)
Mx	1033 (38)	165 (16)
PSA, ng/mL, *n* (%)		
<4	249 (8)	—
4–9	1511 (56)	160 (16)
10–49	961 (35)	389 (38)
≥50	—	454 (44)
Missing	7 (1)	30 (2)
Gleason score, *n* (%)		
≤6	1593 (58)	52 (5)
7	1135 (42)	201 (20)
8–10	—	693 (67)
Missing	—	87 (8)
Primary treatment, *n* (%)^b^		
Conservative	924 (34)	42 (4)
Curative	1601 (59)	336 (32)
Non‐curative	176 (6)	638 (62)
Missing	27 (1)	17 (2)

Abbreviation: PSA, prostate cancer‐specific antigen; SD, standard deviation.

^a^ Aggressive prostate cancer includes T4 or N1 or M1 or Gleason score ≥8 or PSA ≥50 ng/mL.

^b^ Conservative treatment includes watchful waiting and active surveillance; curative treatment includes radical prostatectomy and radiotherapy; non‐curative treatment includes all androgen deprivation therapies (orchiectomy, GnRH agonists and antagonists) and antiandrogens.

**TABLE 3:** Hazard ratios or odds ratios (95% confidence intervals) of prostate cancer incidence and death according to markers of insulin resistance in the full cohort.

Exposure^a^	Incident prostate cancer						Prostate cancer death	
	Non‐aggressive^b^		Aggressive^b^		All			
	*N* men/cases	HR/OR (95% CI)	*N* men/cases	HR/OR (95% CI)	*N* men/cases	HR/OR (95% CI)	*N* men/deaths	HR/OR (95% CI)
Glucose								
Model 1^c^								
Tertile 1	25,460/907	1.00 (ref)	25,460/313	1.00 (ref)	25,547/1307	1.00 (ref)	25,547/150	1.00 (ref)
Tertile 2	21,216/869	0.93 (0.85–1.03)	21,216/356	1.07 (0.92–1.25)	21,304/1313	0.97 (0.89–1.04)	21,304/163	1.03 (0.82–1.29)
Tertile 3	19,743/871	0.93 (0.85–1.03)	19,743/375	1.09 (0.93–1.27)	19,817/1320	0.95 (0.88–1.03)	19,817/160	1.01 (0.79–1.25)
*p* for trend		0.15		0.30		0.23		0.98
Per SD	66,419/2647	0.94 (0.90–0.98)	66,419/1044	1.01 (0.94–1.06)	66,668/3940	0.96 (0.92–0.98)	66,668/473	1.00 (0.87–1.06)
Model 2^d^								
Tertile 1	25,460/907	1.00 (ref)	25,460/313	1.00 (ref)	25,547/1307	1.00 (ref)	25,547/150	1.00 (ref)
Tertile 2	21,216/869	0.95 (0.86–1.04)	21,216/356	1.07 (0.92–1.25)	21,304/1313	0.97 (0.90–1.05)	21,304/163	1.03 (0.82–1.28)
Tertile 3	19,743/871	0.98 (0.90–1.10)	19,743/375	1.12 (0.95–1.31)	19,817/1320	1.00 (0.93–1.09)	19,817/160	1.02 (0.81–1.29)
*p* for trend		0.92		0.17		0.90		0.86
Per SD	66,419/2647	0.97 (0.93–1.02)	66,419/1044	1.03 (0.96–1.10)	66,668/3940	0.99 (0.94–1.01)	66,668/473	1.00 (0.88–1.09)
Per SD, RDR corr^e^		0.93 (0.83–1.05)		1.08 (0.90–1.27)		0.98 (0.86–1.03)		1.00 (0.73–1.24)
TyG index								
Model 1^c^								
Tertile 1	18,851/817	1.00 (ref)	18,851/272	1.00 (ref)	18,927/1165	1.00 (ref)	18,927/121	1.00 (ref)
Tertile 2	18,925/762	0.91 (0.81–0.99)	18,925/269	0.97 (0.81–1.13)	18,984/1114	0.92 (0.85–1.00)	18,984/139	1.13 (0.88–1.44)
Tertile 3	18,888/663	0.76 (0.68–0.84)	18,888/309	1.10 (0.93–1.29)	18,986/1046	0.85 (0.78–0.93)	18,986/147	1.21 (0.95–1.55)
*p* for trend		0.001		0.27		0.001		0.12
Per SD	56,664/2242	0.90 (0.87–0.94)	56,664/850	1.03 (0.97–1.11)	56,897/3325	0.95 (0.91–0.98)	56,897/407	1.06 (0.96–1.16)
Model 2^d^								
Tertile 1	18,851/817	1.00 (ref)	18,851/272	1.00 (ref)	18,927/1165	1.00 (ref)	18,927/121	1.00 (ref)
Tertile 2	18,925/762	0.93 (0.84–1.03)	18,925/269	1.00 (0.83–1.16)	18,984/1114	0.96 (0.88–1.04)	18,984/139	1.13 (0.89–1.45)
Tertile 3	18,888/663	0.82 (0.74–0.92)	18,888/309	1.16 (0.98–1.38)	18,986/1046	0.92 (0.84–1.00)	18,986/147	1.24 (0.97–1.62)
*p* for trend		0.001		0.08		0.05		0.08
Per SD	56,664/2242	0.94 (0.90–1.00)	56,664/850	1.06 (0.99–1.14)	56,897/3325	0.98 (0.94–1.00)	56,897/407	1.07 (0.96–1.18)
Per SD, RDR corr^e^	56,664/2242	0.88 (0.81–1.00)	56,664/850	1.12 (0.98–1.30)	56,897/3325	0.96 (0.88–1.00)	56,897/407	1.14 (0.92–1.39)
Insulin								
Model 1^c^								
Tertile 1	1307/101	1.00 (ref)	1307/42	1.00 (ref)	1330/166	1.00 (ref)	1330/24	1.00 (ref)
Tertile 2	1218/85	0.88 (0.66–1.18)	1218/46	1.15 (0.76–1.74)	1240/153	0.97 (0.78–1.21)	1240/30	1.34 (0.78–2.29)
Tertile 3	1317/95	0.95 (0.72–1.26)	1317/38	0.94 (0.56–1.35)	1328/144	0.96 (0.69–1.07)	1328/25	1.01 (0.57–1.74)
*p* for trend		0.71		0.54		0.19		0.96
Per SD	3842/281	0.94 (0.84–1.07)	3842/126	0.99 (0.82–1.16)	3898/463	0.96 (0.84–1.02)	3898/79	1.00 (0.76–1.17)
Model 2^d^								
Tertile 1	1307/101	1.00 (ref)	1307/42	1.00 (ref)	1330/166	1.00 (ref)	1330/24	1.00 (ref)
Tertile 2	1218/85	0.88 (0.66–1.18)	1218/46	1.17 (0.77–1.79)	1240/153	0.97 (0.77–1.21)	1240/30	1.32 (0.77–2.27)
Tertile 3	1317/95	0.87 (0.72–1.30)	1317/38	0.93 (0.60–1.50)	1328/144	0.90 (0.69–1.11)	1328/25	0.98 (0.54–1.75)
*p* for trend		0.81		0.83		0.27		0.93
Per SD	3842/281	0.95 (0.83–1.08)	3842/126	1.02 (0.85–1.22)	3898/463	0.93 (0.85–1.03)	3898/79	0.99 (0.74–1.17)
HbA1c								
Model 1^c^								
Tertile 1	800/152	1.00 (ref)	800/31	1.00 (ref)	813/196	1.00 (ref)	813/24	1.00 (ref)
Tertile 2	884/131	0.77 (0.60–1.00)	884/44	1.25 (0.87–2.09)	896/187	0.89 (0.70–1.12)	896/35	1.01 (0.69–1.14)
Tertile 3	976/134	0.70 (0.52–0.87)	976/54	1.22 (0.86–2.01)	991/203	0.80 (0.61–0.98)	991/43	1.10 (0.87–1.38)
*p* for trend		0.01		0.23		0.04		0.37
Per SD	2660/417	0.85 (0.75–0.96)	2660/129	1.10 (0.92–1.30)	2700/586	0.92 (0.81–1.01)	2700/102	1.10 (0.97–1.18)
Model 2^d^								
Tertile 1	800/152	1.00 (ref)	800/31	1.00 (ref)	813/196	1.00 (ref)	813/24	1.00 (ref)
Tertile 2	884/131	0.78 (0.60–1.02)	884/44	1.25 (0.82–1.90)	896/187	0.90 (0.71–1.15)	896/35	1.01 (0.66–1.09)
Tertile 3	976/134	0.71 (0.54–0.93)	976/54	1.17 (0.77–1.78)	991/203	0.81 (0.63–1.03)	991/43	1.09 (0.77–1.26)
*p* for trend		0.02		0.51		0.08		0.99
Per SD	2660/417	0.86 (0.75–0.99)	2660/129	1.05 (0.87–1.27)	2700/586	0.90 (0.79–1.01)	2700/102	1.09 (0.95–1.19)
Leptin								
Model 1^c^								
Tertile 1	720/131	1.00 (ref)	720/34	1.00 (ref)	735/180	1.00 (ref)	735/23	1.00 (ref)
Tertile 2	724/108	0.80 (0.60–1.06)	724/31	0.90 (0.55–1.38)	735/150	0.87 (0.60–1.01)	735/26	1.05 (0.80–1.36)
Tertile 3	727/112	0.81 (0.61–1.07)	727/38	1.10 (0.75–1.81)	737/160	0.91 (0.66–1.10)	737/34	1.12 (0.86–1.45)
*p* for trend		0.14		0.48		0.22		0.39
Per SD	2171/351	0.90 (0.79–1.01)	2171/103	1.08 (0.92–1.31)	2207/490	0.94 (0.82–1.03)	2207/83	1.07 (0.90–1.15)
Model 2^d^								
Tertile 1	720/131	1.00 (ref)	720/34	1.00 (ref)	735/180	1.00 (ref)	735/23	1.00 (ref)
Tertile 2	724/108	0.80 (0.60–1.09)	724/31	0.87 (0.54–1.44)	735/150	0.81 (0.60–1.04)	735/26	1.03 (0.77–1.35)
Tertile 3	727/112	0.90 (0.62–1.21)	727/38	1.12 (0.70–1.96)	737/160	0.91 (0.66–1.20)	737/34	1.07 (0.77–1.35)
*p* for trend		0.39		0.54		0.43		0.76
Per SD	2171/351	0.90 (0.79–1.05)	2171/103	1.09 (0.90–1.37)	2207/490	0.92 (0.81–1.07)	2207/83	1.04 (0.85–1.13)

Abbreviations: CI, confidence interval; HR, hazard ratio; OR, odds ratio; RDR, regression dilution ratio; SD, standard deviation.

^a^ All exposures were standardised separately by cohort. Cohort‐specific tertile cut‐points are shown in Table S1.

^b^ Aggressive prostate cancer includes T4 or N1 or M1 or Gleason score ≥8 or PSA ≥50 ng/mL.

^c^ Hazard ratio calculated by use of Cox regression with attained age as time scale, adjusted for baseline age and stratified on cohort and year of birth. Effect sizes for HbA1c and leptin are reported as odds ratios analysed by use of logistic regression due to these factors' origin from a nested case–control study in one of the analysed cohorts (the Västerbotten Intervention Programme). Adjustments were the same as in Cox regression models, with additional adjustment for cohort and year of birth (included as strata variables in Cox models).

^d^ As model 1 with additional adjustment for history of diabetes, country of birth, education, body mass index and smoking status at baseline.

^e^ HRs for glucose and TyG index as a continuous variable were corrected for the regression dilution ratio of 0.40 and 0.50, respectively. Conversion into the uncorrected hazard ratios can be obtained using the equation described in the methods.

In the case‐only survival analysis, glucose and TyG index were shown to be non‐significantly positively related to PCa death (RDR corrected HRs per higher SD, 1.22, 95% CI 1.00–1.49, and 1.24, 95% CI 1.00–1.55) (Table 4). These associations were further pronounced when restricting the analysis to measurements closer than 10 years before the PCa diagnosis (RDR corrected HRs 1.70, 95% CI 1.09–2.70 in 1069 cases/163 PCa deaths, and 1.66, 95% CI 1.10–2.26 in 893 cases/128 PCa deaths, respectively). The exclusion of smokers, obese men, diabetic men and men with comorbidities resulted in strongly attenuated HRs of PCa death by glucose levels and slightly attenuated HRs by TyG index levels (Table S4). The sample size was reduced by 14%–53% in these analyses, and all HR CIs included one. There were no significant associations of insulin, HbA1c and leptin with PCa death; however, case‐only analyses of HbA1c and leptin showed similar effect sizes to those of glucose and TyG index.

**TABLE 4:** Hazard ratios (95% confidence intervals) of prostate cancer death according to markers of insulin resistance in prostate cancer cases.

Exposure^a^	Non‐aggressive prostate cancer^b^		Aggressive prostate cancer^b^		All prostate cancer	
	*N* cases/deaths	HR (95% CI)	*N* cases/deaths	HR (95% CI)	*N* cases/deaths	HR (95% CI)
Glucose						
Model 1^c^						
Tertile 1	1220/32	1.00 (ref)	1220/96	1.00 (ref)	1220/128	1.00 (ref)
Tertile 2	1225/22	0.70 (0.41–1.23)	1225/118	1.24 (0.94–1.63)	1225/140	1.11 (0.87–1.42)
Tertile 3	1246/29	0.91 (0.53–1.57)	1246/117	1.18 (0.89–1.57)	1246/146	1.12 (0.87–1.44)
*p* for trend		0.70		0.26		0.38
Per SD	3691/83	0.84 (0.65–1.09)	3691/331	1.10 (0.97–1.24)	3691/414	1.05 (0.93–1.17)
Model 2^d^						
Tertile 1	1220/32	1.00 (ref)	1220/96	1.00 (ref)	1220/128	1.00 (ref)
Tertile 2	1225/22	0.71 (0.41–1.24)	1225/118	1.31 (0.99–1.73)	1225/140	1.19 (0.93–1.53)
Tertile 3	1246/29	0.90 (0.52–1.58)	1246/117	1.24 (0.93–1.67)	1246/146	1.19 (0.92–1.55)
*p* for trend		0.67		0.15		0.18
Per SD	3691/83	0.85 (0.64–1.08)	3691/331	1.13 (1.00–1.28)	3691/414	1.10 (1.00–1.21)
Per SD, RDR corr^e^	3691/83	0.71 (0.39–1.17)	3691/331	1.29 (1.00–1.67)	3691/414	1.22 (1.00–1.49)
TyG index						
Model 1^c^						
Tertile 1	1022/24	1.00 (ref)	1022/78	1.00 (ref)	1022/102	1.00 (ref)
Tertile 2	1042/18	0.82 (0.45–1.51)	1042/107	1.38 (1.02–1.88)	1042/125	1.24 (0.95–1.64)
Tertile 3	1028/26	1.00 (0.54–1.85)	1028/97	1.68 (1.23–2.30)	1028/123	1.51 (1.14–1.99)
*p* for trend		0.99		0.01		0.01
Per SD	3092/68	1.02 (0.80–1.34)	3092/282	1.21 (1.06–1.37)	3092/350	1.17 (1.05–1.32)
Model 2^d^						
Tertile 1	1022/24	1.00 (ref)	1022/78	1.00 (ref)	1022/102	1.00 (ref)
Tertile 2	1042/18	0.81 (0.44–1.49)	1042/107	1.34 (0.98–1.83)	1042/125	1.25 (0.95–1.65)
Tertile 3	1028/26	0.99 (0.53–1.83)	1028/97	1.45 (1.06–1.99)	1028/123	1.38 (1.04–1.82)
*p* for trend		0.95		0.02		0.03
Per SD	3092/68	1.01 (0.80–1.33)	3092/282	1.14 (1.00–1.30)	3092/350	1.12 (1.00–1.26)
Per SD, RDR corr^e^	3092/68	1.02 (0.66–1.71)	3092/282	1.28 (1.00–1.64)	3092/350	1.24 (1.00–1.55)
Insulin						
Model 1^c^						
Tertile 1	143/3	1.00 (ref)	143/13	1.00 (ref)	143/16	1.00 (ref)
Tertile 2	131/5	1.49 (0.88–3.54)	131/18	1.46 (0.67–2.99)	131/23	1.46 (0.75–2.85)
Tertile 3	133/4	1.23 (0.56–2.35)	133/16	1.35 (0.60–3.02)	133/20	1.25 (0.61–2.59)
*p* for trend		0.85		0.46		0.54
Per SD	407/12	0.61 (0.26–1.40)	407/47	1.27 (0.17–13.11)	407/59	1.00 (0.74–1.30)
Model 2^d^						
Tertile 1	143/3	1.00 (ref)	143/13	1.00 (ref)	143/16	1.00 (ref)
Tertile 2	131/5	1.50 (0.87–3.49)	131/18	1.24 (0.58–2.65)	131/23	1.36 (0.69–2.67)
Tertile 3	133/4	1.15 (0.53–2.28)	133/16	1.11 (0.49–2.51)	133/20	1.09 (0.53–2.24)
*p* for trend		0.92		0.79		0.81
Per SD	407/12	0.59 (0.25–1.40)	407/47	1.00 (0.71–1.30)	407/59	0.98 (0.68–1.18)
HbA1c						
Model 1^c^						
Tertile 1	183/10	1.00 (ref)	183/13	1.00 (ref)	183/23	1.00 (ref)
Tertile 2	175/12	1.49 (0.74–3.09)	175/20	1.53 (0.75–3.09)	175/32	1.33 (0.93–2.12)
Tertile 3	188/6	1.73 (0.87–3.45)	188/34	1.81 (0.91–3.61)	188/40	1.60 (0.98–2.91)
*p* for trend		0.13		0.09		0.05
Per SD	546/28	1.11 (0.87–1.43)	546/67	1.22 (0.98–1.50)	546/95	1.17 (0.95–1.39)
Model 2^d^						
Tertile 1	183/10	1.00 (ref)	183/13	1.00 (ref)	183/23	1.00 (ref)
Tertile 2	175/12	1.32 (0.64–2.72)	175/20	1.01 (0.52–1.90)	175/32	1.05 (0.63–1.75)
Tertile 3	188/6	1.63 (0.80–3.32)	188/34	1.48 (0.69–3.17)	188/40	1.22 (0.91–2.52)
*p* for trend		0.18		0.22		0.51
Per SD	546/28	1.12 (0.84–1.49)	546/67	1.23 (0.94–1.59)	546/95	1.13 (0.90–1.43)
Leptin						
Model 1^c^						
Tertile 1	165/4	1.00 (ref)	165/17	1.00 (ref)	165/21	1.00 (ref)
Tertile 2	139/9	1.77 (0.59–5.26)	139/14	0.67 (0.34–1.30)	139/23	0.90 (0.51–1.55)
Tertile 3	150/9	0.91 (0.23–3.56)	150/23	1.15 (0.58–2.26)	150/32	1.14 (0.62–2.07)
*p* for trend		0.88		0.66		0.68
Per SD	454/22	1.02 (0.61–1.70)	454/54	1.20 (0.88–1.63)	454/76	1.15 (0.89–1.52)
Model 2^d^						
Tertile 1	165/4	1.00 (ref)	165/17	1.00 (ref)	165/21	1.00 (ref)
Tertile 2	139/9	1.77 (0.59–5.26)	139/14	0.84 (0.40–1.72)	139/23	0.98 (0.60–1.97)
Tertile 3	150/9	0.91 (0.23–3.56)	150/23	1.22 (0.57–2.19)	150/32	1.20 (0.68–2.12)
*p* for trend		0.88		0.74		0.78
Per SD	454/22	1.02 (0.61–1.70)	454/54	1.20 (0.89–1.71)	454/76	1.15 (0.87–1.49)

Abbreviations: CI, confidence interval; HR, hazard ratio; SD, standard deviation.

^a^ All exposures were standardised separately by cohort. Cohort‐specific tertile cut‐points are shown in Table S1.

^b^ Aggressive prostate cancer includes T4 or N1 or M1 or Gleason score ≥8 or PSA ≥50 ng/mL.

^c^ Hazard ratio calculated by use of Cox regression with time since diagnosis as time scale, adjusted for age at diagnosis, time since baseline, history of diabetes, country of birth, body mass index, and smoking status, education at the time of diagnosis, and stratified on cohort and year of birth.

^d^ As model 1 with additional adjustment for Charlson comorbidity index, primary treatment for prostate cancer, and prostate cancer risk category.

^e^ HRs for glucose and TyG index as a continuous variable were corrected for the regression dilution ratio of 0.48 and 0.53, respectively. Conversion into the uncorrected hazard ratios can be obtained using the equation described in the methods.

## 4 DISCUSSION

In this pooled prospective study, there was no evidence of insulin resistance markers increasing the risk of PCa. However, higher glucose and TyG index levels were associated with poorer survival from PCa, and these associations were stronger when restricting the analysis to measurements performed <10 years before the PCa diagnosis. The associations were most robust for TyG index, for which the effect size was largely retained after the exclusion of smokers, obese or diabetic men, and men with comorbidities. Insulin, HbA1c and leptin were analysed in much smaller samples, hence with lower statistical power; however, non‐significant poorer PCa‐specific survival was observed for higher levels of HbA1c and leptin.

Results from previous studies of these insulin resistance markers with PCa risk have been inconsistent with results from meta‐analyses showing an overall weak, positive association for plasma insulin levels in prospective studies,^3^ but no association for levels of C‐peptide^2^—a cleavage product of proinsulin and a stable marker of insulin with less measurement error than insulin due to its longer half‐life,^41^ and also no association for leptin levels.^4^ A meta‐analysis of fasting glucose showed an overall negative association with the risk of PCa, which finds some support also for HbA1c levels,^5^ especially in relation to non‐aggressive PCa,^42^ as further supported by our study. A negative association between blood glucose and any and non‐aggressive PCa in some studies may be due to higher PCa screening activity in healthy men,^43^ and similarly, this has also been proposed as explanation for the negative association of obesity with PCa risk.^44^ This is supported by a large, prospective study on the metabolic syndrome and prostate cancer in which high levels of a composite metabolic syndrome score—a condition linked to insulin resistance—were not associated with prostate cancer risk before the PSA screening era, but were associated with a lower risk in the PSA era.^45^ Altogether, evidence from our and other prospective studies speaks against a substantial and causal role of insulin resistance markers in the initiation and development of PCa.

Consistent with other studies,^12^, ^13^, ^14^, ^15^, ^16^, ^17^ higher glucose and TyG index levels were related to a higher risk of PCa death in our study. Patient studies have provided some evidence that men with PCa and T2DM are at an increased risk for biochemical recurrence after radical prostatectomy,^46^, ^47^ further supporting a role of insulin resistance in PCa progression. In our study, we found that higher glucose levels and the TyG index related to a higher risk of PCa death only in the case‐only analysis, and not in the full cohort. This may be explained by that the case‐only analysis inherently captures only survival after PCa diagnosis, whereas the full‐cohort analysis from baseline reflects the association both with PCa incidence and death, which weakens the association for a factor primarily associated with survival. Statistical power was substantially weaker for insulin, HbA1c and leptin in relation to PCa death, but the non‐significant positive effect sizes from these analyses were similar to those of glucose and TyG index on PCa death, which, if replicated, would further support the involvement of insulin resistance or related conditions in PCa progression. However, it also remains unclear whether intervention on insulin resistance markers, before or after PCa diagnosis, could delay PCa progression. This requires investigation also of post‐diagnostic measures of insulin resistance, and ultimately verification by clinical trials.

Biological pathways through which markers of insulin resistance could promote progression to PCa death include both direct and indirect mechanisms. For example, glucose metabolism may have a direct role in PCa progression,^11^ and leptin can stimulate angiogenesis and proliferation of PCa cells.^48^ Insulin resistance further involves increased IGF‐1 levels and chronic inflammation, which may promote PCa progression.^49^, ^50^, ^51^ Overexpression of the IGF‐insulin receptor in PCa patients is related to the progression of PCa by stimulating cell proliferation and promoting tumour growth, by activating the phosphoinositide 3‐kinase (PI3K) and mitogen‐activated protein kinase (MAPK) signalling pathways.^52^, ^53^

This study's strengths include its prospective design, the population‐based origin, the long follow‐up captured in high‐quality national registers^34^, ^35^ and the detailed clinical information on PCa with high validity from the National Prostate Cancer Register of Sweden.^38^ Limitations include the smaller population analysed for insulin, HbA1c and leptin, resulting in less robust results, the lack of post‐diagnostic measures of insulin resistance markers, and the lacking information on antidiabetic medication—a factor that could confound or modify the associations observed in this study.

## 5 CONCLUSION

The findings of this study showed no evidence for an association of insulin resistance markers with the risk of clinically relevant PCa. However, men with higher levels of glucose and the TyG index had poorer PCa‐specific survival, and this association was also indicated in the results of the smaller analytic samples of HbA1c and leptin. These findings may indicate that insulin resistance plays a role in the progression of PCa, which requires further investigation in observational and clinical studies.

## AUTHOR CONTRIBUTIONS

**Sylvia H. J. Jochems:** Conceptualization (equal); data curation (equal); formal analysis (lead); investigation (equal); methodology (equal); writing – original draft (equal). **Josef Fritz:** Investigation (equal); methodology (equal); writing – review and editing (equal). **Christel Häggström:** Investigation (equal); methodology (equal); writing – review and editing (equal). **Pär Stattin:** Data curation (equal); investigation (equal); writing – review and editing (equal). **Tanja Stocks:** Conceptualization (equal); data curation (equal); funding acquisition (lead); investigation (equal); methodology (equal); writing – original draft (equal).

## FUNDING INFORMATION

This study was supported by the Swedish Cancer Society (2017/475, 2017/1019, and 201,033 PjF), the Swedish Research Council (2018–02825), the World Cancer Research Fund International (IIG_FULL_2020_025), the Crafoord Foundation (20200546), the Swedish Prostate Cancer Federation and the Cancer Research Foundation at the Department of Oncology, Malmö University Hospital, Sweden.

## CONFLICT OF INTEREST STATEMENT

The authors have no competing interests to declare.

## ETHICS APPROVAL STATEMENT

The ethics committee at Lund University, Sweden, approved the study (No. 2016/564).

## Supporting information


Table S1.
**Table S2**.
**Table S3**.
**Table S4**.

## Data Availability

Data are available from the corresponding author conditional on permission from the involved cohort committees and national registers.
