# Supplementary material for: Prediagnostic markers of insulin resistance and prostate cancer risk and death: A pooled study
Source: Cancer Med. 2023 Apr 27;12(12):13732–44. doi: 10.1002/cam4.6004 (PMC10315749; doi:10.1002/cam4.6004)
Supplement: Supplementary file 1 — Table S1. Table S2. Table S3. Table S4. [file CAM4-12-13732-s001.docx]

**Supplementary material**

Pre-diagnostic markers of insulin resistance and prostate cancer risk and death: a pooled study

Sylvia H.J. Jochems, Josef Fritz, Christel Häggström, Pär Stattin, Tanja Stocks

Table S1. Tertile cut-points of insulin resistance markers according to cohort

| **Exposure** | **VIP** | **MONICA** | **MDCS** | **MPP** |
| --- | --- | --- | --- | --- |
| Glucose, mmol/L | 5.3; 5.7 | 5.2; 5.5 | 4.9; 5.3 | 4.8; 5.2 |
| TyG index^a^ | 8.4; 8.8 | 8.4; 8.7 | 8.3; 8.7 | 8.3; 8.7 |
| Insulin, mIU/L | - | - | 5; 8 | 6; 9 |
| HbA1c, % | 4.3; 4.6 | - | 4.6; 5.0 | - |
| Leptin, ng/ml | 3.2; 4.8 | - | 1.8; 2.8 | - |

VIP – Västerbotten Intervention Programme, MONICA – Northern Sweden Monica Study, MDCS – Malmö Diet and Cancer Study, MPP – Malmö Preventive Project.

^a^ TyG index was calculated as ln[triglycerides (mg/dL) × plasma glucose (mg/dL)/2].

Table S2. Hazard ratios and odds ratios (95% confidence intervals) of prostate cancer incidence and death per standard deviation increase of HbA1c and leptin in the Malmö Diet and Cancer Study

|  | **Incident prostate cancer** | | | **Prostate cancer death** | | |
| --- | --- | --- | --- | --- | --- | --- |
| **Exposure** | **N men/cases** | **HR (95% CI)^a^** | **OR (95% CI)^b^** | **N men/deaths** | **HR (95% CI)^a^** | **OR (95% CI)^b^** |
| HbA1c | 2165/280 | 0.98 (0.87-1.15) | 0.95 (0.84-1.13) | 2165/32 | 1.12 (0.79-1.68) | 1.07 (0.92-1.21) |
| Leptin | 1754/221 | 0.99 (0.85-1.16) | 0.97 (0.83-1.17) | 1754/24 | 1.00 (0.51-1.45) | 1.02 (0.87-1.21) |

HR – Hazard ratio, CI – confidence interval, OR – odds ratio.

^a^ Hazard ratio calculated by use of Cox regression with attained age as time scale, adjusted for baseline age, history of diabetes, country of birth, education, body mass index and smoking status, and stratified on year of birth.

^b^ Odds ratio calculated by use of logistic regression adjusted for baseline age, year of birth, country of birth, history of diabetes, education, body mass index and smoking status.

Table S3. Correlation coefficients^a^ between the insulin resistance markers and BMI

|  | **Glucose** | **TyG index** | **Insulin** | **HbA1c** | **Leptin** |
| --- | --- | --- | --- | --- | --- |
| BMI | 0.27 | 0.36 | 0.32 | 0.26 | 0.39 |
| Glucose |  | 0.36 | 0.33 | 0.28 | 0.36 |
| TyG index |  |  | 0.35 | 0.30 | 0.35 |
| Insulin |  |  |  | 0.26 | 0.48 |
| HbA1c |  |  |  |  | 0.25 |

Spearman’s partial rank correlation coefficient of cohort-specific z-transformed insulin resistance markers and BMI, adjusted for age.

All p-values for the correlations are <0.01.

Table S4. Hazard ratios (95% confidence intervals) of prostate cancer death according to per standard deviation increase in glucose levels and TyG index in prostate cancer cases

| **Exposure** | **N cases/deaths** | **HR (95% CI)^a^** |
| --- | --- | --- |
| Glucose |  |  |
| Excluding smokers (current and former) | 1745/177 | 1.03 (0.70-1.41) |
| Excluding obese men (BMI ≥30 kg/m^2^) | 3370/374 | 1.04 (0.82-1.34) |
| Excluding men with diabetes | 3567/398 | 1.11 (0.88-1.39) |
| Excluding men with severe comorbidities | 3497/390 | 1.11 (0.88-1.41) |
| Excluding men with any comorbidities | 3215/346 | 1.00 (0.77-1.27) |
| TyG index |  |  |
| Excluding smokers (current and former) | 1443/147 | 1.24 (0.91-1.84) |
| Excluding obese men (BMI ≥30 kg/m^2^) | 2852/315 | 1.14 (0.91-1.43) |
| Excluding men with diabetes | 2979/337 | 1.20 (0.96-1.50) |
| Excluding men with severe comorbidities | 2949/331 | 1.20 (0.94-1.50) |
| Excluding men with any comorbidities | 2719/294 | 1.08 (0.84-1.37) |

HR – hazard ratio, CI – confidence interval, BMI – body mass index.

^a^ Hazard ratio calculated by use of Cox regression with time since diagnosis as time scale, adjusted for age at diagnosis, time since baseline, history of diabetes, country of birth, body mass index, and smoking status, education at the time of diagnosis, Charlson comorbidity index, primary treatment for prostate cancer, and prostate cancer risk category, and stratified on cohort and year of birth. The hazard ratios were corrected for regression dilution ratio by 0.48 for glucose and 0.53 for TyG index.
